# Role of Mean Platelet Volume in the Prognosis of Locally Advanced Gastric Cancer: A Tertiary Cancer Center Experience

**DOI:** 10.7759/cureus.9109

**Published:** 2020-07-10

**Authors:** Manjunath K V, Pavan Jonnada, Sai Kiran N, Ali Anwar

**Affiliations:** 1 Surgical Oncology, Kidwai Memorial Institute of Oncology, Bangalore, IND; 2 Surgical Oncology, Kidwai Memorial Institute of Oncology, Bengaluru, IND

**Keywords:** mean platelet volume, gastric cancer, locally advanced, nodal disease, stage

## Abstract

Introduction

Mean platelet volume (MPV) is an inflammatory marker suggesting the activation of platelets. Many studies observed an association between MPV and cancer spread and metastasis. Hence, we have conducted a retrospective study to find the role of MPV as a prognostic marker in locally advanced gastric cancer.

Materials and methods

The present study included a retrospective review of 149 patients with gastric cancer who had neoadjuvant chemotherapy followed by surgery. MPV was obtained and then statistically analyzed to find an association between tumor (T), node (N), and overall stage as per the American Joint Committee on Cancer (AJCC) staging system, using Statistical Package for the Social Sciences (SPSS) software (IBM Corp., Armonk, NY).

Results

In our study, we observed that MPV values were significantly high in N+ disease (OR 3.794 (95% CI 1.903 - 7.563); p-value 0.0001), higher T stage (OR for >T2 3.692 (95% CI 1.876 - 7.266); p-value 0.0001), and advanced stage (OR 7.708 (95% CI 3.258 - 18.237); p-value 0.0001) of gastric cancer.

Conclusions

MPV is an inflammatory marker that correlates with nodal disease and aids in the staging and prognostication of locally advanced gastric cancer. This inexpensive, convenient marker can aid in the risk stratification of locally advanced gastric cancer.

## Introduction

Gastric cancer is the fifth most common cancer and the third leading cause of cancer death [1]. The survival rates of Stages I, II, III, and IV were 80%-90%, 70%, 35%-54%, and 16%, respectively [2]. Despite improvements in screening and advancements in treatment, gastric cancer often presents at an advanced stage. The presentation of early gastric cancer at a stage at which it is resectable is observed in only 33% of cases [3]. Hence, it is crucial to diagnose the disease at an early stage, which can have a significant improvement in survival with timely treatment.

Several tumor markers have been used in the diagnosis, prognosis, and early detection of gastric cancer. The most frequent tumor markers used for the early detection of gastric cancer include carcinoembryonic antigen (CEA), the carbohydrate antigens (CAs) CA19-9, CA72-4, CA125, CA24-2, and CA50, and pepsinogen and α-fetoprotein (AFP) [4]. Even so, the sensitivity of the markers used remains unsatisfactory and so far, none of them is unique for diagnostics or prognostic for gastric cancer [4-5]. Therefore, the identification of novel diagnostic and prognostic markers for gastric cancer is crucial.

Several factors, such as dietary, infectious, and genetic factors, are suspected to contribute to the development of gastric cancer [6]. Furthermore, in previous studies, it has been shown that gastric cancer represents an underlying inflammation-driven malignancy transformation [7].

Mean platelet volume (MPV) is a routinely determined parameter by complete blood count (CBC) analyzers [8]. It indicates the average size of platelets, the platelet production rate, and stimulation [9]. MPV has been revealed as a marker of inflammation in hepatocellular carcinoma and pancreatic adenocarcinoma [10].

The aim of this study is to examine whether MPV would be a useful inflammatory marker and assess whether MPV could predict nodal status and appropriate cut-off value. Besides, we investigated the relationship between MPV levels and cancer stage in gastric cancer patients.

## Materials and methods

We retrospectively investigated gastric cancer patients who underwent surgical resection at Kidwai Memorial Institute of Oncology between November 2017 and July 2019. Metastatic gastric cancer patients who underwent upfront surgery without neoadjuvant chemotherapy were excluded from the study. One-hundred forty-nine gastric cancer patients were included in the study. The staging of cancer was made according to tumor-node-metastases (TNM) classification and classified through the American Joint Committee on Cancer (AJCC) recommendations. The preoperative data were obtained from the recorded computerized database. The study was undertaken after institutional review board review clearance and with appropriate consent.

Blood analysis

About 5-7 ml, blood was collected from a peripheral vein and was collected into sterile ethylenediaminetetraacetic acid (EDTA) tubes. Blood samples were taken in the morning to minimize the impact of circulating hormones (circadian rhythm). Within 30 minutes after sample collection, an analysis of blood parameters was done by using a hematology analyzer. MPV was, therefore, obtained and used in the consequent analysis. The MPV chosen for the study is the MPV at the time of diagnosis. 

The histopathological characteristics included the histological type and tumor grade. The laboratory variables were hemoglobin (g/fL) and hematocrit (%),MPV (fL). Blood samples for laboratory analysis were been taken after eight to 12 hours of fasting.

Statistical analysis

Statistical analyses were performed with the Statistical Package for the Social Sciences (SPSS) software (IBM Corp., Armonk, NY). All parameters were expressed as means and standard deviation. The chi-square test was used to compare the categorical variables. The paired sample test was used to compare the preoperative and postoperative variables. A receiver-operating characteristic (ROC) curve analysis was performed to identify the optimal cut-off values of the MPV level. A p-value of less than 0.05 was considered statistically significant.

## Results

We studied 149 patients of non-metastatic gastric cancer; there were 105 males and 44 females. The age of the patients was between 22 and 75 years and the mean age was 54.6. The baseline characteristics are presented in Table 1.

**Table 1:** Baseline demographic characteristics TNM: tumor-node-metastases

Baseline characteristics of the study participants	
Number of subjects	149
Age range	22 - 75 years
Mean age	54.6 years
Sex	
Male	106 (28.9%)
Female	43 (71.1%)
Mean platelet volume – fl (mean)	9.87 fL
TNM staging	
Stage 1	48
Stage 2	56
Stage 3	45
Histology	
Intestinal	118
Diffuse	31

Laboratory tests showed that gastric cancer patients enrolled in this study had MPV values ranging between 7.8 and 11.5 fl; the mean MPV value was 9.87 fl.

**Figure 1:**
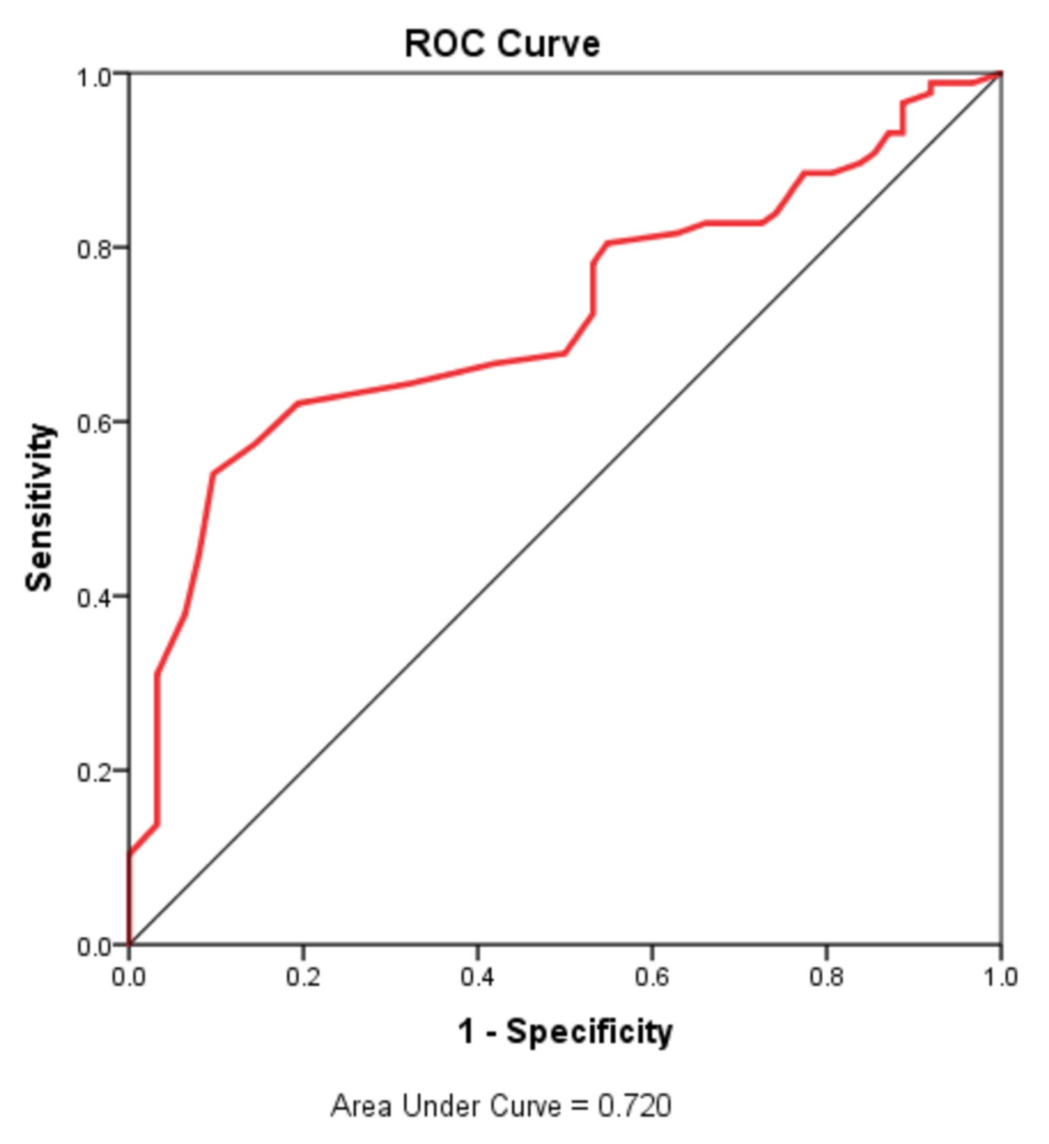
ROC curve analysis showing MPV cut-off ROC: receiver operating characteristic; MPV: mean platelet volume

ROC curves were constructed to allot optimal cut-off values of MPV associated with lymph node positivity in gastric cancer patients. Results were significant (p-value = 0.0001). The area under the curve was 0.720 for MPV (Figure 1). The analysis showed that MPV higher than 10.25 indicates the positivity of lymph nodes with 62% sensitivity and 80% specificity.

**Figure 2:**
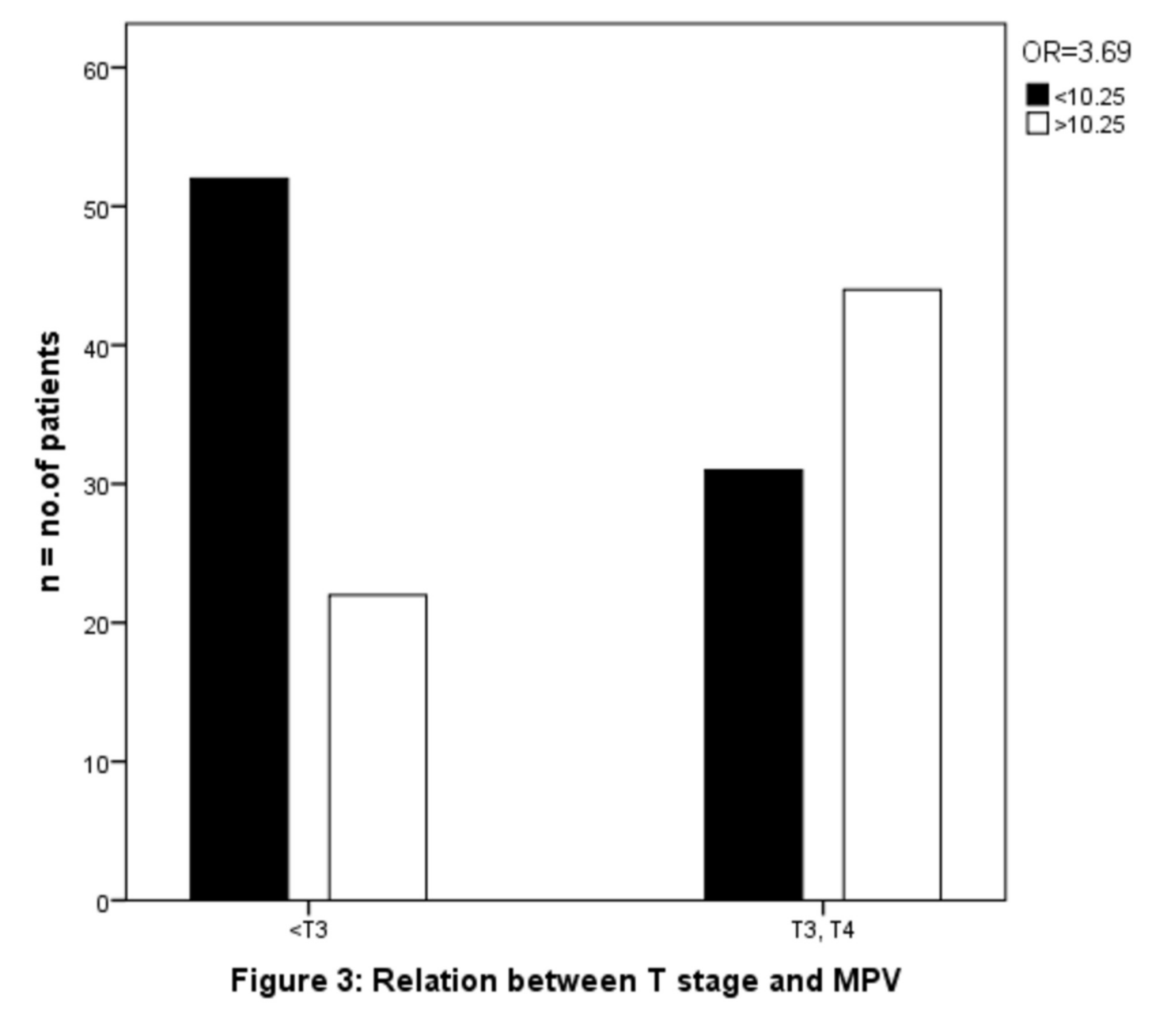
Relation between T stage and MPV T: tumor; MPV: mean platelet volume

**Figure 3:**
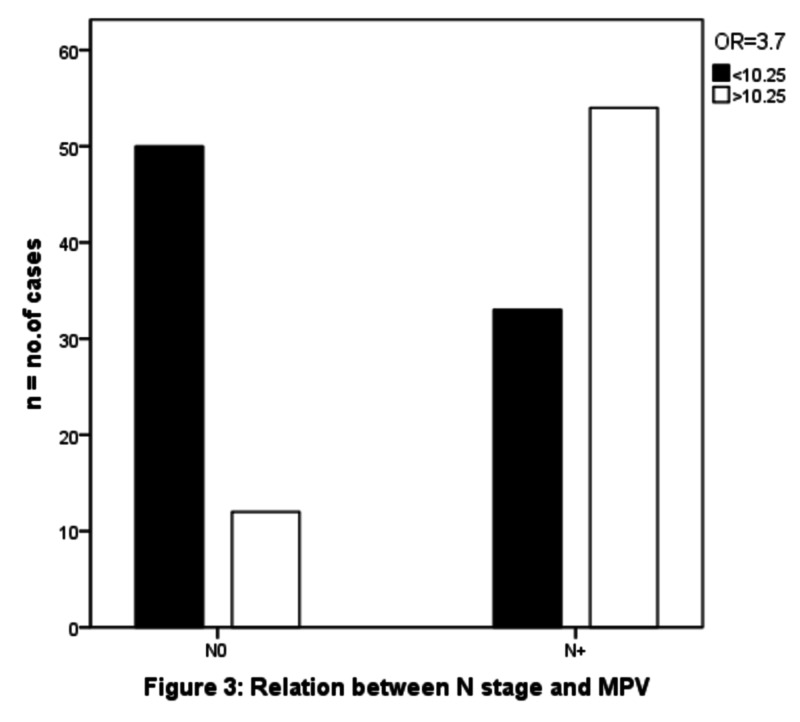
Relation between N stage and MPV N: node; MPV: mean platelet volume

**Figure 4:**
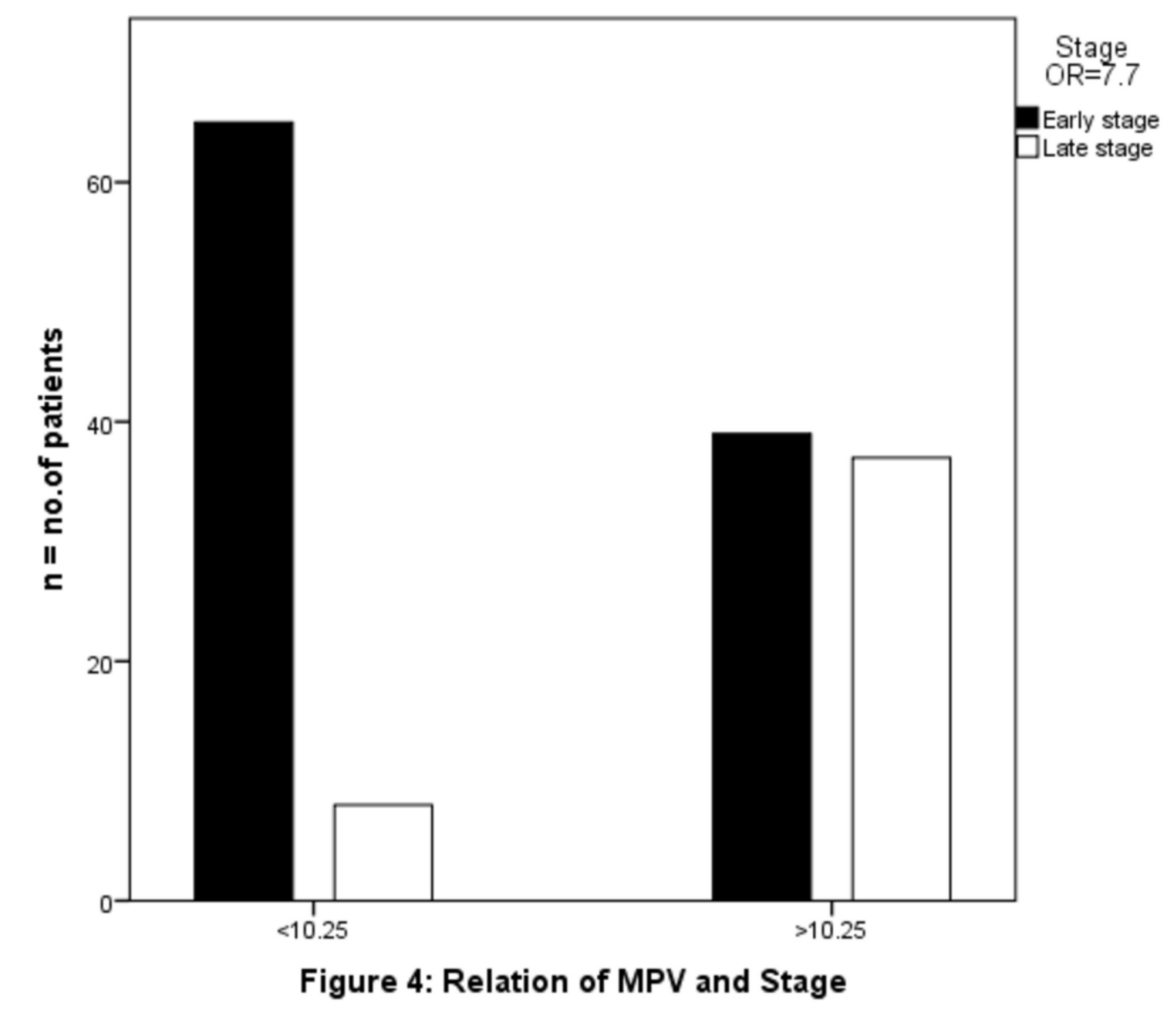
Relation between stage and MPV MPV: mean platelet volume

The patients were separated into two groups according to median preoperative MPV: low (<10.2) MPV and high (≥10.2) MPV. There was no significant association was found between high MPV values and sex (OR 0.8 (95% CI 0.428 - 1.769) p-value - .700). Advanced age was not found to be associated with increasing MPV (OR 1.006 (95% CI 0.517-1.958) p-value .98). We observed that greater MPV values were associated with a higher T stage (OR 3.692 (95% CI 1.876- 7.266) p-value .0001) as shown in Figure 2, with a higher N stage (OR 3.794 (95% CI 1.903 - 7.563) p-value .0001) as shown in Figure 3 and with advanced stages (OR 7.708 (95% CI 3.258- 18.237) p-value .0001), as shown in Figure 4. There was a significant negative association noted between elevated MPV and the diffuse type of histology of gastric cancer (OR 0.345 (95% CI 0.147 - 0.812) p-value .012), as shown in Table 2.

**Table 2:** Relationship between MPV and demographic and clinical parameters T: tumor; N: node; MPV: mean platelet volume

	No.of patients	Low MPV ( <10.2)(n)	High MPV (>10.2)(n)	Chi square	p-value	Odds Ratio (95% CI)
Gender				0.149	.700	0.8 (0.42-1.76)
Male	106	53	53			
Female	43	20	23			
Age				.0001	0.985	1.006 (0.51-1.95)
< 50 years	55	27	28			
> 50 years	94	46	48			
T stage				14.819	.0001	3.962 (1.87-7.26)
T1, T2	74	48	26			
T3, T4	75	25	50			
N stage				14.936	.0001	3.794 (1.90-7.56)
N0	62	42	20			
N +	87	31	56			
Histology				6.241	.012	0.345 (0.14-0.81)
Intestinal	118	64	54			
Diffuse	31	9	22			
Stage				25.139	.0001	7.708 (3.25-18.23)
Stage I, II	104	65	39			
Stage III	45	8	37			

## Discussion

The present study established the role of MPV as an indicator of the status of nodal disease and, thereby, the prognosis of locally advanced resectable gastric cancer. Our results show that MPV can be a future prognostic marker that can aid in detecting the presence of nodal disease and correlate with the T-N staging of the AJCC staging system and, hence, over all the stages of the disease.

MPV increases with the activation or hyper-aggregation of platelets, reflecting the functional state and volume of platelets in the blood circulation. MPV serves as a negative or positive acute-phase reactant and is a marker of inflammation [11]. Cancer-associated inflammation is involved in the development and dissemination of multiple types of tumors [12]. The cascade responsible for the occurrence of cancer involves mainly two steps. First, tumorigenesis is mainly due to deoxyribonucleic acid (DNA) damage and defects in the repair mechanism by the production of reactive oxygen species (ROS) by inflammatory cells. Second, inflammatory cells produce cytokines, chemokines, and cell adhesion molecules, which are responsible for metastasis [13]. Several studies have confirmed the role of MPV in the inflammatory cascade, and the study by Khode et al. showed that high MPV levels are associated with a high risk of acute myocardial infarction when compared with healthy controls [14].

The elevation of MPV gained significance in malignancies as reported in several studies. Li et al. suggested that higher MPV levels are significantly observed in colon cancer patients when compared to healthy controls [15]. Kilincalp et al. suggested that MPV could be used as an early marker of the diagnosis of gastric cancer and can be used to monitor healthy subjects. Their study also showed that MPV levels were markedly raised in gastric cancer patients [16]. However, the study by Zhi-Yuan Yun et al. contradicted these findings, raising doubts regarding the role of MPV as an early diagnostic marker in gastric cancer patients [17].

Sun SY et al. suggested that low MPV was significantly associated with locally advanced esophageal carcinoma and may serve in the screening and risk stratification of locally advanced esophageal carcinomas [18]. Our study showed that increased MPV is significantly associated with a higher T and N stage and, thereby, might be helpful in risk stratification of the disease. Shen et al. showed that higher preoperative MPV values were significantly associated with lower survival rates for patients of resectable gastric cancer patients [19]. We investigated whether higher MPV could assess the nodal disease and, to our knowledge, this is the first study demonstrating the role of MPV as a maker of locally advanced disease.

Various studies have used different cut-offs for MPV in various cancers. In our study, an MPV cut-off of 10.25 indicated the positivity of lymph nodes with 62% sensitivity and 80% specificity based on the ROC analysis. A similar level of cut-off was used in other contemporary studies on gastric cancer [19].

Our study has its limitations, as it is a retrospective series based on case records. A major drawback of this investigation is the limited detail available for each case. Furthermore, all patients underwent neoadjuvant chemotherapy and those with a favorable response underwent surgery. This may indicate the favorable biology of the tumors and may not completely reflect the role of MPV.

However, the present study indicates an association of the MPV level and with the local invasion and prognosis of gastric cancer. Even though MPV is also a non-specific marker, this non-invasive, well-situated, and inexpensive biomarker may be an addition to the present biomarkers and a benefit to the prognosis and risk stratification of locally advanced gastric cancer.

## Conclusions

Mean platelet volume is an inexpensive, convenient marker that correlates with nodal disease and aids in staging, prognostication, and risk stratification of locally advanced gastric cancer. The MPV cut-off value of 10.25 predicts nodal metastases and a higher T stage and thus provides the stage of the disease. The incorporation of MPV into current prognostic factors may provide additional information to stratify the disease and may provide an opportunity for further investigation into the natural history and prognosis of the disease.
